# Examiner workload comparison: three structured oral examination formats for the European diploma in anaesthesiology and intensive care

**DOI:** 10.1080/10872981.2024.2364990

**Published:** 2024-06-07

**Authors:** Mikhail Dziadzko, Andrey Varvinskiy, Rodolphe Di Loreto, Hugues Scipioni, Bazil Ateleanu, Markus Klimek, Joana Berger-Estilita

**Affiliations:** a Department of Anesthesia, Intensive Care and Pain Management, Hospices Civils de Lyon, Hôpital de la Croix Rousse, Lyon, France; b Research on Healthcare Performance (RESHAPE) U1290-INSERM, Université Claude Bernard Lyon 1, Lyon, France; c South Devon Healthcare NHS Foundation Trust, Department of Anesthesia and Intensive Care, Torquay, UK; d European Society of Anaesthesiology and Intensive Care, Examinations Office, Brussels, Belgium; e European Society of Anesthesiology and Intensive Care, Examinations Committee, Brussels, Belgium; f Department of Anaesthesia, University Hospital of Wales, Cardiff, UK; g Department of Anaesthesiology, Erasmus University Medical Centre, Rotterdam, Netherlands; h Institute for Medical Education, University of Bern, Bern, Switzerland; i Hirslanden Hospital Group, Institute of Anaesthesiology and Intensive Care, Salem Spital, Bern, Switzerland; j CINTESIS - Centre for Health Technology and Services Research, Faculty of Medicine, Porto, Portugal

**Keywords:** Anaesthesiology, continued education, evaluation, examiner taskload, online assessment, structured oral examination

## Abstract

The COVID-19 pandemic triggered transformations in academic medicine, rapidly adopting remote teaching and online assessments. Whilst virtual environments show promise in evaluating medical knowledge, their impact on examiner workload is unclear. This study explores examiner’s workload during different European Diploma in Anaesthesiology and Intensive Care Part 2 Structured Oral Examinations formats. We hypothesise that online exams result in lower examiner’s workload than traditional face-to-face methods. We also investigate workload structure and its correlation with examiner characteristics and marking performance. In 2023, examiner’s workload for three examination formats (face-to-face, hybrid, online) using the NASA TLX instrument was prospectively evaluated. The impact of examiner demographics, candidate scoring agreement, and examination scores on workload was analysed. The overall NASA TLX score from 215 workload measurements in 142 examiners was high at 59.61 ± 14.13. The online examination had a statistically higher workload (61.65 ± 12.84) than hybrid but not face-to-face. Primary contributors to workload were mental and temporal demands, and effort. Online exams were associated with elevated frustration. Male examiners and those spending more time on exam preparation experienced a higher workload. Multiple diploma specialties and familiarity with European Diploma in Anaesthesiology and Intensive Care exams were protective against high workload. Perceived workload did not impact marking agreement or examination scores across all formats. Examiners experience high workload. Online exams are not systematically associated with decreased workload, likely due to frustration. Despite workload differences, no impact on examiner’s performance or examination scores was found. The hybrid examination mode, combining face-to-face and online, was associated with a minor but statistically significant workload reduction. This hybrid approach may offer a more balanced and efficient examination process while maintaining integrity, cost savings, and increased accessibility for candidates.

## Introduction

The outbreak of the COVID-19 pandemic has triggered significant transformations in the academic teaching of medicine, leading to the rapid implementation of remote education and the use of alternative methods for assessing knowledge, notably online examinations [1]. Effective learning and training have been facilitated by various communication tools like visual media, digitised content, and web platforms. At the same time, trainees and trainers have identified different learning and knowledge assessment barriers, including resource scarcity, technical issues, high costs, and inadequate user training [2].

A recent systematic review has demonstrated an enhanced confidence in using virtual environments to assess graduate and postgraduate medical knowledge assessment. This primarily applied to Objective Structured Clinical Examinations (OSCEs), profoundly disrupted by the COVID-19 restrictions and social distancing [3] It also applied to Structured Oral Examinations (SOEs), widely adopted as a standardised assessment method for evaluating various physician competencies in postgraduate training [4].

Assessing a candidate’s performance is a complex cognitive process. It includes forming initial impressions, active listening, detecting and selecting relevant performance elements, interacting with peer examiners, processing, assimilating and categorising responses in the working memory, and making a final judgement [5].

Whilst the candidate’s performance mainly influences the examiner workload [5,6], environmental and organisational factors related to the examination process can also have an impact. These factors encompass the physical presence of the examiner or candidate, the need to interact with software or hardware, and external distractions like logistical issues during breaks. In turn, the marginal task load (very high or very low) may influence the clarity of decision-making, creating the potential for errors and poor examiner’s performance [6,7].

Current research has demonstrated a high students’ acceptability and satisfaction with online remote education models [8]. However, little is known about examiners’ experience from the implementation of online evaluation of medical knowledge, particularly regarding task load, which different examination modes may modify. Until now, no research has focused on measuring the examiner’s task load in the context of these varied SOE formats.

The European Diploma in Anaesthesiology and Intensive Care (EDAIC) is a two-step examination designed to enable standardised evaluation of the professional knowledge of candidates who have received training in various countries worldwide. It comprises written multiple choice-based questions covering basic science (Part 1) and a SOE for contextual evaluation of basic and clinical knowledge (Part 2) [9].

In response to the challenges posed by the COVID-19 epidemic, the European Society of Anaesthesiology and Intensive Care (ESAIC) developed three formats for Part 2 SOE. These formats include a fully online assessment conducted via Zoom® Platform which was introduced in 2020, a hybrid format exam combining a plenary session for examiners with remote candidates (introduces in 2022), and a traditional face-to-face (F2F) format, running since the creation of the EDAIC.

With this study, we aim to assess the global task load experienced by examiners following these different formats of Part II SOEs for EDAIC. We hypothesise that the online examination type will generate a lower workload compared to the traditional F2F one. Additionally, we analysed the examiner’s task load structure and explored its relationships with candidate performance and the agreement in marking among examiner pairs across different examination formats.

## Methods

### Study design

We conducted a prospective observational study to assess the examination workload of active examiners of Part II EDAIC during the year 2023. This study adhered to the ethical standards outlined in the Declaration of Helsinki and complied with the Data Protection Acts, thereby ensuring ethical compliance and the protection of participant data.

### Settings and participants

Independently from the examination type, each candidate for Part II EDAIC passes four successful SOE containing two basic science papers and two clinical papers. Each paper contains five questions, the duration of assessment is five minutes per question. Each SOE is performed by a pair of examiners assessing the candidate’s response using a three-point marking system (0-fail to answer, 1 -borderline answer and 2 – correct, structured and detailed answer) per question. Each examiner scores the candidate and submits results via an online evaluation system. Scores from each pair of examiners are summarised, giving the SOE score (maximum of 20), and the final examination note is the sum of SOE scores (maximum of 80).

Four unique examiner pairs are required per candidate (one examiner interacts with a candidate only once). Each pair usually examines up to 12 candidates per day. A detailed description of examination procedure is available on the web-page of ESAIC [10].

The ESAIC organised the 2023 exam calendar in three formats: F2F, Hybrid, and Online examinations. F2F examinations require the physical presence of both candidates and examiners in the same premises simultaneously. The hybrid examination consists of the physical presence of examiner pairs and the remote online participation of candidates. Online examination implies full online participation both for examiners and candidates. ESAIC Examination Office staff organises all necessary interactions between examiners and candidates.

EDAIC Part II examiners are qualified physicians, members of ESAIC, most of themselves diplomates of EDAIC, in active professional practice worldwide. All active current year 2023 examiners were invited to participate in the study. The participation was voluntary, and the protocol with its purposes was presented to the potential participants during the pre-examination briefing one day before examination.

### Outcomes and measurements

The primary outcome was the examiner’s perceived global workload in different examination formats at the end of each examination day, measured with The NASA Task Load Index (NASA-TLX) [11]. This index quantifies subjective mental workload across six dimensions: mental demand, physical demand, temporal demand, performance, effort, and frustration. The evaluation assesses the relative importance (load) of six dimensions (dominant importance of the paired items – for example, Effort vs Mental Demands) experienced during the SOE. The intensity for each dimension was presented with a linear scale ranging from 0 (minimal) to 10 (maximal), culminating in a comprehensive load-weighted NASA TLX score ranging from 0 (no task load) to 100 (maximum task load). Designed for the aeronautic industry, the NASA-TLX was already used to evaluate the perceived workload in clinical settings [12] and medical education [13]. NASA TLX is considered low for values inferior to 10, medium for 10–29, somewhat high for 30–49, high for 50–79, and very high for scores 80 and more [14].

**Figure 1.**
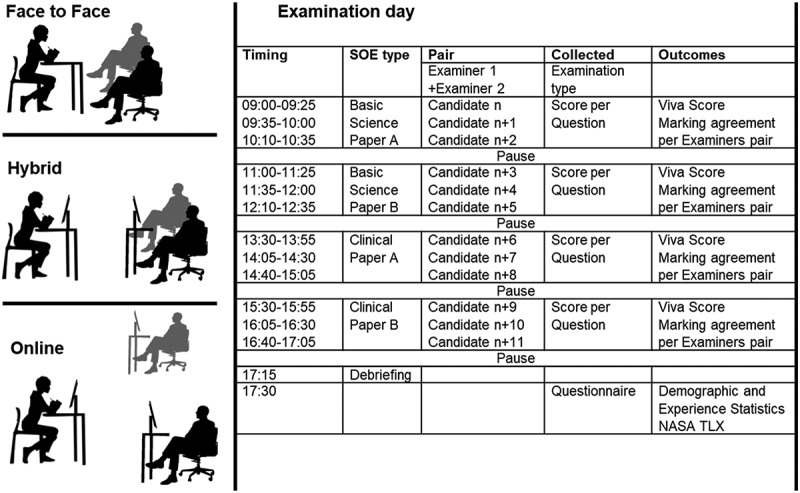
Study Outline.SOE – Structured Oral Examination; n is the order number for the candidate

Secondary outcomes included the relationships between measured task load and demographic and professional characteristics of examiners (gender, years in practice, previous experience in EDAIC examining, supplementary qualifications, time spent to prepare for EDAIC examination), agreement in candidate scoring, and per-SOE examination score (Figure 1).

### Data collection

Data for the primary outcome measurement and demographic characteristics were collected with the NASA-TLX instrument and a questionnaire (Appendix, Supplement 1), implemented using the online tool SurveyMonkey® (SurveyMonkey Inc., San Mateo, CA, USA). At the end of each examination day, examiners who agreed to participate were invited to complete the electronic survey using a link in their MyESAIC account. Examination type, candidate performance, and information for examiner pairing were extracted from the Ortrac Evaluation System® (Orzone, Gothenburg, Sweden). The final dataset was managed using JMP Pro 13.0 (SAS Institute Inc., Cary, N.C., USA).

### Sample size calculation and statistics

The sample size was calculated for the superiority trial. We hypothesised that the meaningful difference in workload is 30%. We assumed an arbitrary standard deviation of 10% for each group, and the expected difference between groups was set at 20%. To achieve a statistical power of 80% at a 95% confidence level, a sample size of approximately 38 participants per group is required to detect a meaningful significant difference of 30 points on the continuous scale (ranging from 0 to 100) with a standard deviation of 10. This sample size estimation is based on a two-sided hypothesis test.

Categorical variables were described as absolute (n), and relative frequencies (%). Continuous variables were systematically assessed for normal distribution using the Shapiro-Wilks test and presented as mean ± SD or median [25–75% interquartile range, IQR] accordingly. Due to the nature of the data, we employed the chi-square test or Fisher exact test for categorical variables, and ANOVA with Tukey’s HSD Test for multiple comparisons or Kruskal Wallis and Steel Dwass test.

To evaluate the agreement between a pair of examiners, we employed kappa statistics calculated for each SOE examination. This approach allowed us to assess the level of agreement on a per-case basis, providing information on grading performance. Multivariate methods with correlation analysis were used to explore the relationships of NASA TLX components and an overall score, expressed as rho and 95% confidence interval. Multiple linear regression analysis was implemented to explore the factors potentially influencing task load. Results are expressed as estimates, standard error, and p-value. An a priori probability of less than 0.05 was considered statistically significant. All calculations were performed using JMP Pro 13.0 (SAS Institute Inc., Cary, N.C., USA).

## Results

During the 2023 examination year, we gathered 215 task load measurements from 142 examiners for 1027 candidates. Although the number of examinations was unequally distributed between all three examination types, no statistically significant differences were found regarding demographic and professional characteristics between groups (Table 1).

**Table 1.** Demographic and professional characteristics of examiners.

	Total	F2F	Hybrid	Online
Number of Responses, n(%)	215 (100%)	38 (18%)	73 (34%)	104 (48%)
Unique Examiners, n(%)	142 (100%)	25(18%)	45 (32%)	72 (50%)
Female examiners, n(%)	56 (39%)	8 (32%)	23 (51%)	56 (39%)
Number of unique examinees	1027 (100%)	254 (25%)	359 (35%)	414 (40%)
Examinees per Examiner	7	10	8	6
Experience in Anaesthesiology, years	18.5 [13 to 26]	18 [15 to 23]	18 [12 to 27]	19 [14 to 25]
EDAIC Part II Exam days performed in the last year				
0	17 (11%)	5 (20%)	3 (7%)	9 (12%)
1–3	57 (39%)	8 (32%)	23 (51%)	26 (34%)
4–9	60 (41%)	11 (44%)	18 (40%)	31 (40%)
>9	13 (9%)	1 (4%)	1 (2%)	11 (14%)
Holding of another specialty or subspecialty				
None	20 (14%)	7 (28%)	5 (11%)	8 (11%)
Intensive Care Medicine	75 (53%)	11 (44%)	23 (51%)	41 (57%)
Education (MME)	15 (11%)	2 (8%)	6 (13%)	7 (10%)
Pain Medicine	12 (8%)	2 (8%)	4 (9%)	6 (8%)
Prehospital & Emergency	12 (8%)	1 (4%)	5 (11%)	6 (8%)
Management (MBA)	3 (2%)	0 (0%)	1 (2%)	2 (3%)
Other	2 (1%)	1 (4%)	0 (0%)	1 (1%)
Paediatrics	2 (1%)	1 (4%)	1 (2%)	0 (0%)
Internal Medicine	15 (11%)	0 (0%)	0 (0%)	1 (1%)
Another diploma besides EDAIC, yes	67 (47%)	12 (48%)	21 (47%)	34 (47%)
Examiner of another European diploma, yes	21 (15%)	2 (8%)	7 (16%)	12 (17%)
Hours spent for Examination preparation				
1h	5 (4%)	1 (4%)	1 (2%)	3 (4%)
2h	19 (13%)	4 (16%)	4 (9%)	11 (15%)
3h	24 (17%)	1 (4%)	10 (22%)	13 (18%)
>3h	94 (66%)	19 (76%)	30 (67%)	45 (62%)

F2F – face-to-face; EDAIC – European Diploma in Anaesthesia and Intensive Care; MME – Master of Medical Education; MBA – Master of Business Administration.

The mean overall taskload was high at 59,61 ± 14.13, more important for online examination type, significant comparing to hybrid (5.3 points difference, 95%CI [0.27 to 10.36], *p* = 0.0363), and comparable to the F2F type. Seventy six percent of examiners expressed high workload (≥50), with no significant difference across examination type, however in Hybrid examination the proportion of examiners who expressed very high workload was significantly lower comparing to other examination types (*p* = 0.0484). All results are presented in the Table 2.

**Table 2.** Perceived examiners’ task load.

	Total n = 215	F2F n = 38	Hybrid n = 73	Online n = 104
NASA TLX	59,61±14.13	59,97±16,4	56,38±14,23	61,65±12,84
Examiners with TLX≥50, n(%)	164 (76%)	29 (76%)	51 (70%)	84 (81%)
TLX <10 (Low), n(%)	0 (0%)	0 (0%)	0 (0%)	0%)
10 ≤ TLX < 30 (Medium), n(%)	6 (3%%)	2 (5%)	4 (5%)	0%)
30 ≤ TLX < 50 (Somewhat high), n(%)	45 (21%)	7 (18%)	18 (25%)	20 (19%)
50 ≤ TLX < 80 (High), n(%)	146 (68%)	25 (66%)	49 (67%)	72 (69%)
TLX ≥ 80 (Very high), n(%)	18 (8%)	4 (11%)	2 (3%)	13 (12%)

F2F – face-to-face, TLX – taskload index

**Figure 2.**
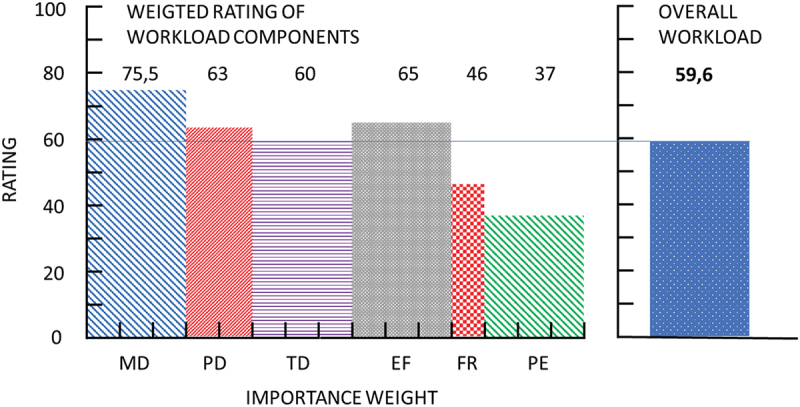
Measures components of perceived workload.Vertical axis is a NASA TLX component rating, horizontal axis is a weight of each component. MD – Mental Demand; PD – Physical Demand; TD – Temporal Demand; EF – Effort; FR – frustration; PE – Performance

Figure 2 illustrates the overall weighted rating of measured workload components, highest for mental demand and effort; and lowest for frustration and performance. Correlation analysis on the global taskload and its components was positive for all dimensions except frustration score (rho = 0.05 95% CI [−0,08 to 0,18], *p* = 0.46), more confirming the highest impact of mental, temporal demand and effort (all significant with *p* < .05, Supplemental Table 1).

There was no statistically significant difference between all three examination types for all weighted components of NASA TLX score except for Frustration Score, that was significantly higher comparing to the online and hybrid examinations (11 points difference, 95%CI [0.09 to 22], *p* = 0.0476) (Supplemental Table 2).

The mean NASA TLX score was significantly higher in male examiners 62 ± 13 vs 57 ± 15 (females), *p* = 0.0115; there was no effect of the duration of professional experience in anaesthesiology, however the number of examinations performed in the previous year was associated with decreased workload, with weak exposition effect − 0.5 score decrease per three examinations increment (*p* < .0001). Holding more than one specialty or subspecialty, other than general anaesthesiology, is associated with lower taskload: 59 ± 14 vs 66 ± 13, *p* = 0.0101. Having another diploma besides the EDAIC (national or international diploma) was also associated with a lower task load: 57 ± 15 vs 62 ± 13, *p* = 0.0109. No difference in task load was found for those who examined for another European Diploma compared to sole EDAIC examiners. A higher task load was associated with the time needed for the examination preparation: the increment of 1 hour spent for preparation is associated with 4.7 points of NASA TLX increase, statistically significant (*p* < 0.0001).

Kappa statistics calculation was available for 380 paired SOE (papers) examinations (190 examinees), which included 120 examiners, with a median value of 0.55 [IQR 0.29 to 1], with statistically different but without meaningful significance across types of examination (Supplemental Table S3).

There were no relations between NASA TLX score and kappa agreement nor examination score for all type of examinations. In the multivariable analysis, the hybrid examination mode was associated with lower taskload (Estimate −1.13 Standard Error 0.56, *p* = 0.0438), no association with agreement examination score was found.

## Discussion

Our study found that EDAIC Part II examiners consistently experienced a high workload (59 out of 100) across all examination types. Notably, the workload was significantly higher for the online examination type than for other formats. A substantial number of examiners reported a very high workload for both F2F and online examination formats, whilst hybrid examinations seemed to result in a lower perceived workload. The key contributors to this overall task load were mental demand, temporal demand, and effort, which were consistently high across all examinations.

When looking at differences between examination types, frustration emerged as the primary factor contributing to the NASA TLX score difference, and this frustration was particularly associated with the online examination format.

Male examiners and those who spent more time preparing for upcoming examinations tended to perceive a higher workload. However, examiners with experience across multiple specialties and diplomas and those familiar with EDAIC examinations seemed to be better equipped to handle the workload, experiencing a lower perceived task load.

Surprisingly, perceived workload did not appear to impact marking agreement during examinations or the actual examination scores across all formats. No U-shape patterns were found when exploring relationships between workload and examiner’s performance [15,16].

Contrary to our initial hypothesis, we found a statistically significant difference indicating a higher workload for the online examination type compared to the face-to-face and hybrid formats. Although this difference is, in practice, not substantial (less than a 30% change), it challenges the assumption that online examinations inherently reduce examiner task load. Online exams were also associated with lower per-SOE assessment scores compared to other exam formats, but perceived workload, measured by NASA TLX scores, did not show a clear association with grading agreement or overall exam scores. The strong assessment value of online examinations was confirmed regardless of the type of questions, students’ year, academic discipline, or class size [17], therefore, we cannot draw any inferences regarding the impact of the examination type on marking.

In healthcare, studies on teleworking have highlighted various advantages and disadvantages [18]. Notably, issues related to technology, reduced interaction with colleagues, and the blending of home and work responsibilities were identified as the most significant contributors to the negative effects of the full-time teleworking model [19]. Similar challenges may apply to online examinations, particularly when considering the frustration experienced by examiners. Our analysis revealed that frustration was central to the NASA TLX score difference between examination types, strongly associated with the online examination format. This suggests that challenges specific to online exams, such as technical issues or examiner unfamiliarity with the technology, may significantly contribute to examiner frustration. These findings underscore the importance of addressing these issues to improve the overall experience of online examinations for both examiners and candidates.

Interestingly, the hybrid examination mode, which combines real-world interactions with some online elements, was associated with a reduction in perceived workload. While the reduction was statistically significant, the actual difference was relatively small, less than 30%. Nevertheless, this hybrid approach could offer a more balanced and efficient way of conducting examinations while maintaining their integrity and effectiveness. The frustration score for the hybrid examination was similar to that of the F2F, suggesting examiners’ resilience to potential technical issues associated with remote technologies. Furthermore, beyond just reducing workload, the hybrid model has the potential to provide advantages such as improved grading quality and cost savings for candidates who don’t have to incur travel expenses. This aspect could enhance accessibility and equity in examination processes, making them more inclusive and less burdensome for both examiners and candidates.

To our knowledge, no published studies have focused on how teleworking affects specialists in medical education. Applying the universal social relationships model, we stress the importance of holistic understanding of examiner workload. Strategies to tackle frustration and enhance the user experience in online examinations should be considered when designing assessment.

Our study has several limitations. We did not capture all the different aspects influencing an individual’s perception of workload, and the sample size was not designed for multivariable analysis. No candidate’s opinion was gathered to reinforce the analysis of examination mode differences. The NASA Task Load Index, a subjective tool for assessing workload, could be influenced by individual biases and emotional states, which were not measured. The absence of a control group and the lack of randomisation among examiners across different examination modes might introduce confounding factors that could skew the study’s outcomes.

Additionally, the varying levels of experience and expertise among the examiners could affect the results, as more experienced examiners might perceive the process as less demanding. The comfort level of examiners with technology, especially in online and hybrid modes, is another factor likely to impact their reported workload. External factors, such as internet connectivity and the physical setup of examination environments, were not controlled for, which could influence workload perceptions in different modes. The voluntary nature of participation in the study could lead to selection bias, attracting examiners who may already have particular concerns about workload. Lastly, since the study is set within the context of European medical education, its findings might not be directly transferable to other educational and cultural environments; however, the EDAIC examiners (along with candidates) represent not only different practice countries but also different ethnic groups and nations.

## Conclusion

The SOE for EDAIC Part II is associated with constantly high workload. The hybrid but not the online examination mode contributes to workload decrease, offering the potential to balance efficiency, fairness, and accessibility in the examination process. The lack of a significant impact on grading quality, despite high workloads, is a testament to the examiners’ commitment but also a reminder of the need to support these professionals to sustain this level of performance.

Further interventional research addressing the optimisation of examiner’s workload in different operational environments may be interesting using NASA TLX tool as a standard for workload evaluation.

## Supplementary Material

Supplemental Material
