# Supplementary material for: Examiner workload comparison: three structured oral examination formats for the European diploma in anaesthesiology and intensive care
Source: Med Educ Online. 2024 Jun 7;29(1):2364990. doi: 10.1080/10872981.2024.2364990 (PMC11164053; doi:10.1080/10872981.2024.2364990)
Supplement: Supplemental Material [file ZMEO_A_2364990_SM0598.docx]

**Appendices**

**Supplement 1**

**Part A: Demography and Experience questions**

| **Question** | **Answers** | |
| --- | --- | --- |
| Years of experience in Anaesthesiology | number | |
| Do you hold another specialty/subspecialty? | yes/no | if yes : which one? |
| Do you have another diploma besides the DESAIC? | yes/no | if yes : which one? |
| Are you an examiner of another European Diploma? | yes/no | if yes : which one? |
| EDAIC Part II Exam days performed in the last year | 0 \| 1-3 \| 4-9 \| >9 | |
| For this exam, how many hours did you use for question preparation? | 1h \| 2h \| 3h \| >3h | |

**Part B: NASA TLX test**

This evaluation assesses the relative importance of six factors in determining how much workload you experienced during examination. You asked to choose which of the paired items (for example, Effort vs Mental Demands) was more important to your workload experience during examination.

The following factors are included:

| Dimension | Question |
| --- | --- |
| Mental Demand | How much mental and perceptual activity was required? Was the task easy or demanding, simple or complex? |
| Physical Demand | How much physical activity was required? Was the task easy or demanding, slack or strenuous? (ex. technical tasks, material use, software manipulation, etc.) |
| Temporal Demand | How much time pressure did you feel due to the pace at which the tasks or task elements occurred? Was the pace slow or rapid? |
| Performance | How successful were you in performing the task? How satisfied were you with your performance? |
| Effort | How hard did you have to work (mentally and physically) to accomplish your level of performance? |
| Frustration | How irritated, stressed, and annoyed versus content, relaxed, and complacent did you feel during the task? |

**TEST 1**

Circle the scale title that represents the more important contributor to workload of the examination you performed in this day

| Effort  --- or ---  Performance | Temporal Demand  --- or ---  Frustration | Temporal Demand  --- or ---  Effort | Physical Demand  --- or ---  Frustration | Performance  --- or ---  Frustration |
| --- | --- | --- | --- | --- |
| Physical Demand  --- or ---  Temporal Demand | Physical Demand  --- or ---  Performance | Temporal Demand  --- or ---  Mental Demand | Frustration  --- or ---  Effort | Performance  --- or ---  Mental Demand |
| Performance  --- or ---  Temporal Demand | Mental Demand  --- or ---  Effort | Mental Demand  --- or ---  Physical Demand | Effort  --- or ---  Physical Demand | Frustration  --- or ---  Mental Demand |

**TEST 2**

**What was the intensity for each dimension during the examination**

| Dimension | Question | Scale |
| --- | --- | --- |
| Mental Demand | How much mental and perceptual activity was required? Was the task easy or demanding, simple or complex? | │ I │ I │ I │ I │ I │ I │ I │ I │ I │ I │  Very Low Very High |
| Physical Demand | How much physical activity was required? Was the task easy or demanding, slack or strenuous? (ex. technical tasks, material use, software manipulation, etc.) | │ I │ I │ I │ I │ I │ I │ I │ I │ I │ I │  Very Low Very High |
| Temporal Demand | How much time pressure did you feel due to the pace at which the tasks or task elements occurred? Was the pace slow or rapid? | │ I │ I │ I │ I │ I │ I │ I │ I │ I │ I │  Very Low Very High |
| Performance | How successful were you in performing the task? How satisfied were you with your performance? | │ I │ I │ I │ I │ I │ I │ I │ I │ I │ I │  Very Low Very High |
| Effort | How hard did you have to work (mentally and physically) to accomplish your level of performance? | │ I │ I │ I │ I │ I │ I │ I │ I │ I │ I │  Very Low Very High |
| Frustration | How irritated, stressed, and annoyed versus content, relaxed, and complacent did you feel during the task? | │ I │ I │ I │ I │ I │ I │ I │ I │ I │ I │  Very Low Very High |

**Supplement 2**

Supplemental Table 1 Correlation matrix of the overall taskload and its components

| Global Score | Score Component | Correlation | Confidence interval | p-value |
| --- | --- | --- | --- | --- |
| NASA TLX | Mental Demand Score | 0,46 | 95%CI [0,35 to 0,56] | <,0001 |
| NASA TLX | Physical Demand Score | 0,22 | 95%CI [0,09 to 0,34] | 0,001 |
| NASA TLX | Temporal Demand Score | 0,46 | 95%CI [0,35 to 0,56] | <,0001 |
| NASA TLX | Effort Score | 0,44 | 95%CI [0,33 to 0,54] | <,0001 |
| NASA TLX | Frustration Score | 0,05 | 95%CI [-0,08 to 0,18] | 0,4583 |
| NASA TLX | Performance Score | 0,3 | 95%CI [0,18 to 0,42] | <,0001 |

TLX - taskload

Supplemental Table 2 A NASA TLX Components across different examination types

| Weighted Scores | Total  n=215 | F2F  n=38 | Hybrid  n=73 | Online  n=104 |
| --- | --- | --- | --- | --- |
| Mental Demand | 75,5±32 | 73±35 | 72±36 | 78±28 |
| Physical Demand | 63±33 | 64±34 | 59±30 | 65±34 |
| Temporal Demand | 60±42 | 61±43 | 55±43 | 64±41 |
| Effort | 65±35 | 64±35 | 65±36 | 67±34 |
| Frustration | 46±35 | 39±30 | 39±33 | 50±35^*^ |
| Performance | 37±38 | 41±44 | 35±36 | 36±37 |

F2F-face to face; data are presented as means and standard deviation

There was no statistically significant difference between all three examination types for all weighted components of NASA TLX score except Frustration Score. A one-way ANOVA revealed a statistically significant difference in Frustration component between groups (F(2, 114) = 3.55, p = 0.0321). Tukey’s HSD Test for multiple comparisons yield the mean value of Frustration Score significantly different between Online Examination Type and Hybrid (11 points difference, 95%CI [0.09 to 22], p = 0.0476).

Supplemental Table 3 Kappa agreement in different examination types

| Weighted Scores | Total | F2F | Hybrid | Online |
| --- | --- | --- | --- | --- |
| Agreement Kappa | 0.55  [IQR 0.29 to 1] | 0.62*  [IRQ 0.55 to 1] | 0.55  [IRQ 0.25 to 1] | 0.55  [IQR 0.29 to 1] |

F2F-face to face; data are presented as medians and interquartile range; * p= Steel Dwass Nonparametric Comparison for All Pairs test - higher agreement in F2F examination (difference 0.7, z-score 2.6, p= 0.0248).
